# Dietary Leucine Supplementation Restores Serum Glucose Levels, and Modifying Hepatic Gene Expression Related to the Insulin Signal Pathway in IUGR Piglets

**DOI:** 10.3390/ani9121138

**Published:** 2019-12-13

**Authors:** Jingfei Zhang, Wen Xu, Hongli Han, Lili Zhang, Tian Wang

**Affiliations:** College of Animal Science and Technology, Nanjing Agricultural University, No. 6, Tongwei Road, Xuanwu District, Nanjing 210095, China; zhangjingfei@njau.edu.cn (J.Z.); xuwenable@126.com (W.X.); 2018105060@njau.edu.cn (H.H.); zhanglili@njau.edu.cn (L.Z.)

**Keywords:** leucine, serum glucose, insulin resistance, IUGR

## Abstract

**Simple Summary:**

Intrauterine malnutrition may compromise the size and structure of fetal organs and tissues, which leads to lower birth weight and a slower rate of growth after weaning. Intrauterine growth restriction/retardation (IUGR) impairs pancreas function, resulting in the decreased glucose levels in serum. Leucine, one of branched chain amino acids, is an essential amino acid and the substrate of protein synthesis. Leucine also acts as a major regulator of hormone signal transduction, like insulin. Dietary branched chain amino acids or leucine have beneficial effects on the glucose metabolism and glycogen synthesis of muscle. Leucine supplementation improves the insulin sensitivity in liver and muscle and then influences the systemic glucose homeostasis. However, it is still unclear whether leucine supplementation would alter insulin sensitivity in IUGR neonatal piglets. Our results showed that dietary leucine supplementation restored serum glucose concentrations, increased insulin and creatinine concentrations, and enhanced protein kinase adenosine monophosphate-activated γ 3-subunit and glucose transporter type 2 expression. These findings suggest that leucine might play a positive role in hepatic lipid metabolism and glucose metabolism in IUGR.

**Abstract:**

This study aimed to investigate the effects of leucine with different levels on the insulin resistance in intrauterine growth restriction/retardation (IUGR) piglets. Thirty-two weaned piglets were arranged in a 2 × 2 factorial design and four treatments (n = 8) were as follow: (1) normal weaned piglets fed a basal diet (CONT), (2) IUGR weaned piglets fed a basal diet (IUGR), (3) normal weaned piglets fed a basal diet with the addition of 0.35% l-leucine (C-LEU), and (4) IUGR fed a basal diet with the addition of 0.35% l-leucine (I-LEU) for a 21-days trial. The results showed that compared to the IUGR group, the I-LEU group had higher final body weight and body weight gain, higher serum glucose concentrations, and higher serum insulin concentrations (*p* < 0.05). The gene expression of phosphatidylinositol 3-kinase p110 gamma, protein kinase adenosine monophosphate-activated γ 3-subunit, glycogen synthase kinase-3 alpha, and glucose transporter type 2 were increased in the I-LEU group as compared to the IUGR group (*p* < 0.05). It was concluded that dietary leucine supplementation restored serum glucose concentrations, increased insulin and creatinine concentrations, and enhanced protein kinase adenosine monophosphate-activated γ 3-subunit and glucose transporter type 2 expression, suggesting that leucine might play a positive role in hepatic lipid metabolism and glucose metabolism in IUGR.

## 1. Introduction

Intrauterine growth restriction/retardation (IUGR), as characterized by lower birth weight, is one of the most common complications during pregnancy and is associated with high morbidity and mortality in neonatal humans and livestock, especially in multiparous animals (e.g., pigs) [1,2]. IUGR impairs the organ development, compromises the size and structure of organs, and leads to dysfunction of the corresponding organs [3]. In livestock production, beyond the congenital malformations and genetic defects, IUGR is also induced by many factors, including environmental insult, maternal and uteroplacental malnutrition, and disease infection [4,5]. A number of studies documented that IUGR is associated with early compensatory growth and later occurrence of the abnormity of glucose and insulin metabolism in pigs [6,7,8]. However, the effects of IUGR on the glucose and insulin metabolism are controversial. In suckling piglets, IUGR increased serum glucose and insulin levels, indicating an increased risk of insulin resistance [9]. In weaning piglets, IUGR exerted adverse effects on the concentrations of serum glucose and insulin [10]. Whether IUGR affects glucose and insulin metabolism in a dynamic manner is still unclear.

Amino acids play an important role in the regulation of animal growth and development. Branched-chain amino acids (BCAA), consisting of leucine, isoleucine, and valine, are not synthesized in mammals and play important roles in physiological and nutritional regulation [11]. Leucine, one of the branched chain amino acids, is an essential amino acid and building block of protein [12,13]. Numerous studies showed that leucine not only stimulates the protein synthesis but also acts as a major regulator in glucose metabolism and insulin signaling [14,15]. Leucine supplementation affected the susceptibility to high-fat diet-induced obesity and promoting insulin signaling in insulin-target tissues in rats [16]. Long-term high-protein diet could induce insulin resistance and lead to increased serum glucose concentrations and decreased glucose output [17]. Leucine induced activation of the insulin signal transduction pathway in an insulin-resistant model in mice [18]. Oral leucine supplementation prevented hyperglycemia-induced insulin resistance in mice fed a high-fat diet [19]. Eller et al. showed that there was an improvement in glucose homeostasis and inhibition of insulin resistance by the administration with leucine [20]. Leucine functions as a positive regulator of insulin secretion [21]. Thus, leucine administration stimulates insulin secretion and increases serum glucose levels.

Moreover, leucine is capable of regulating serum glucose levels through the insulin signal pathway. Liang et al. found that optimal dietary leucine level improved the ability of glycometabolism, including improving glucose transporter 2 (GLUT2) in juvenile fish [22]. In obese rats, chronic leucine supplementation altered the expression of hepatic insulin receptor substrate (IRS) and protein kinase B (Akt) in liver, which were two key genes responsible for insulin sensitivity [12]. Supplementation of leucine decreased the gene expression of hepatic glucose-6-phosphatase (G-6-P), suggesting reduced gluconeogenesis in liver [19]. Dietary branched chain amino acids or leucine had beneficial effects on the glucose metabolism and glycogen synthesis of muscle [20,23]. Thus, it is believed that leucine has direct effects on the insulin signaling pathways in liver and muscle and then influences the systemic glucose homeostasis.

Several studies show that there is a possible relationship between IUGR and the disorder of glucose and insulin metabolism in later life. Pancreas is a potential target organ for leucine and also involved in the control of glucose homeostasis. IUGR impairs pancreas function, resulting in decreased glucose and insulin levels [24]. To optimize the nutritional intervention of these IUGR piglets, renewed focus on the post-weaning stage of dietary is needed, such as leucine. Our previous study showed that leucine supplementation could improve growth performance in IUGR piglets [25]. However, it is still not known whether leucine supplementation would restore the abnormal changes of serum glucose and insulin levels and the gene expression of insulin signaling pathway. Therefore, the present study was carried out to investigate the disturbance of glucose and insulin metabolism induced by IUGR and evaluate the effects of leucine supplementation on serum glucose and insulin levels, and the insulin signal pathway in both normal and IUGR piglets.

## 2. Materials and Methods

### 2.1. Ethical Approval

All experimental procedures were reviewed and approved by Nanjing Agricultural University Institutional Animal Care and Use Committee, China, and pigs were cared for according to the guidelines for experimental animals of the Ministry of Science and Technology (Beijing, China; No. NJAU20180512-1).

### 2.2. Animals and Experimental Design

Pregnancy sows with similar parity (third or fourth) were chosen and fed the same commercial diet. At the time of parturition (day 114 (SD = 1) of gestation), thirty-two piglets from 16 litters were classified at birth as either normal or IUGR on the basis of their birth weight according to previous studies [25]. In the present study, the average birth weights of normal and IUGR piglets were 1.52 (SD = 0.06) and 0.87 (SD = 0.04) kg, respectively. The experiment was conducted on 16 pairs of normal and IUGR piglets (Duroc × [Landrace×Yorkshire]) weaned at 14 d of age. The weaned normal and IUGR piglets were fed a basal diet (1.45% l-leucine) or a basal diet with the addition of 0.35% l-leucine for a 21-day trial. The dose of 0.35% for leucine concentration in the present study was determined on the basis of previous reports [26,27].

The basal diets were formulated according to National Research Council requirements, and their compositions are presented in Table 1.

Four treatments were arranged in a 2 × 2 factorial design with leucine supplementation (with the addition of 0% or 0.35% l-leucine) and birth weight (normal or IUGR). Each treatment consisted of eight replicates of one piglet (four males and four females) per replicate. The treatments were as follows: (1) normal weaned piglets fed a basal diet (CONT group), (2) IUGR weaned piglets fed a basal diet (IUGR group), (3) normal weaned piglets fed a basal diet with the addition of 0.35% l-leucine (C-LEU group), and (4) IUGR weaned piglet fed a basal diet with the addition of 0.35% l-leucine (I-LEU group).

Piglets were housed individually in pens (1 × 0.6 m) with a slatted plastic floor in an environmentally controlled room. During the whole experiment, all piglets had free access to feed and water. Pigs and feeders were weighed on experiment day (ED) 0, ED7, ED14, and ED21 to calculate body weight gain (BWG), average daily protein intake, average daily fat intake, and average daily energy intake.

### 2.3. Sample Collection

After overnight fasting, peripheral venous blood was collected taken by jugular venipuncture on ED0, ED7, ED14, and ED21. The blood samples were allowed to stand for 20 min and then followed by centrifugation at 4000×  *g* for 10 min at 4 °C. Serum were obtained and stored at −80 °C until analysis.

At 35 d of age, eight piglets per treatment were euthanized, and liver samples were collected. The obtained liver samples were snap-frozen in liquid nitrogen and stored at −80 °C until further analysis.

### 2.4. Determination of Serum Glucose, Insulin and Leucine Concentrations

Serum glucoses concentrations were measured with the commercial kits (Jiancheng Bioengineering Institute of Nanjing, Nanjing, China). Serum insulin concentrations were measured by enzyme-linked immunosorbent assay (ELISA) with a commercial kit (Beijing Beifang Biotech Research Institute, Beijing, China) according to the manufacturer’s instructions. The homeostasis models of assessment for the insulin resistance index (HOMA-IR) were calculated as follows: HOMA-IR = (fasting glucose (mmol/L) × fasting insulin (μU/mL))/22.5.

The concentrations of serum leucine were analyzed using a high-performance liquid chromatography method (Venusil-AA HPLC column; Agela Technologies, Newark, DE, USA) according to our previous study [26].

### 2.5. Determination of Liver Glycogen Levels

Liver samples were homogenized (W/V = 1:9) in an ice-cold sodium chloride solution through the use of Ultra-Turrax homogenizer (Tekmar Co., Cincinatti, OH, USA) and centrifuged at 3500× *g* for 10 min at 4 °C. The supernatant was stored at −80 °C for determination of liver glycogen levels. The liver glycogen levels were measured with a commercial kit (Jiancheng Bioengineering Institute of Nanjing, Nanjing, China) according to the manufacturer’s instructions.

### 2.6. Determination of Serum Total Cholesterol, Triglyceride and Creatinine Concentrations

Serum total cholesterol, triglyceride, and creatinine concentrations were measured with the commercial kits (Jiancheng Bioengineering Institute of Nanjing, Nanjing, China) according to the manufacturer’s instructions.

### 2.7. Gene Expression Analysis

Total RNA was extracted from liver samples using the Trizol reagent (TaKaRa Biotechnology Co., Dalian, China), according to the manufacturer’s instructions. The concentration of RNA in the final preparations was measured with a Nanodrop ND-2000c spectrophotometer (Thermo Fisher Scientific, Camden, USA). Reverse transcription was immediately performed using the Primer-ScriptTM reagent Kit (Takara Biotechnology Co., Dalian, China).

The expression of genes in liver was measured using real-time quantitative PCR (RT-qPCR) with SYBR Premix Ex Taq II kit (Tli RNaseH Plus; Takara Biotechnology) and an ABI 7300 Fast Real-Time PCR detection system (Applied Biosystems). The 20 μL reaction system included 2 μL of cDNA template, 0.4 μL of ROX reference dye (50×), 10 μL of SYBR Premix Ex Taq (2×), 0.4 μL of each forward and reverse primer, and 6.8 μL of double-distilled H_2_O. The RT-qPCR cycling conditions were as follows: 95 °C for 30 s, followed by 40 cycles of 95 °C for 5 s and 60 °C for 30 s. All of the samples were run in triplicate, and the mRNA expression level of genes were calculated using the 2^−ΔΔCt^ method and normalized to the value of the reference gene β-actin. The final result of each target gene expression was expressed as the percentage of the CONT group. Primer sequences are shown in Table 2.

### 2.8. Statistical Analysis

The results are presented as mean and standard error mean (n = 8). Data were analyzed by two-way ANOVA using the general linear models (GLM) of SPSS 17.0 (SPSS, Inc., Chicago, IL, USA). The main effects of the model included leucine supplementation levels (0 and 0.35 %) and birth weight (normal and IUGR piglets). The differences between treatments were further analyzed by one-way ANOVA using a Tukey’s post hoc analysis test (*p* < 0.05).

## 3. Results

### 3.1. Effects of Leucine on Growth Performance in IUGR Piglets

The effects of dietary leucine on the body weight and feed intake of piglets are shown in Table 3. Final body weight (FBW), BWG, average daily protein intake, and average daily fat intake and average daily energy intake of the 21 d experimental period were significantly affected by birth weight (*p* < 0.05). The interaction between birth weight and leucine supplementation was significant for FBW, BWG, average daily protein intake, and average daily fat intake and average daily energy intake of the 21 d experimental period (*p* < 0.05). Compared to the CONT group, piglets in the IUGR group had lower FBW and BWG (*p* < 0.05). Compared to the IUGR group, piglets in the I-LEU group had higher FBW and BWG (*p* < 0.05). Between the 14th and 21st day of the experimental period, BWG was significantly affected by birth weight and leucine supplementation (*p* < 0.05).

### 3.2. Effects of Leucine on Serum Glucose, Insulin, and Leucine Concentration in IUGR Piglets

The serum glucose, insulin and leucine concentrations are shown in Table 4. Serum glucose levels on ED14 and ED21 were significantly affected by birth weight and leucine supplementation (*p* < 0.05). The interaction between birth weight and leucine supplementation was significant for serum glucose levels on ED14 and ED21 (*p* < 0.05). Compared to those in the CONT group, piglets in the IUGR group had lower serum glucose concentrations (*p* < 0.05). Compared to those in the IUGR group, piglets in the IL group had higher serum glucose concentrations (*p* < 0.05).

Serum insulin concentrations on ED7, ED14, and ED21 were significantly affected by birth weight and leucine supplementation (*p* < 0.05). The interaction between birth weight and leucine supplementation was significant for serum insulin concentrations on ED7, ED14, and ED21 (*p* < 0.05). Compared to those in the CONT group, piglets in the IUGR group had much lower serum insulin concentrations (*p* < 0.05). Compared to those in the IUGR group, piglets in the I-LEU group had higher serum insulin concentrations (*p* < 0.05).

HOMA-IR on ED7, ED14, and ED21 were significantly affected by birth weight and leucine supplementation (*p* < 0.05). The interaction between birth weight and leucine supplementation was significant for HOMA-IR on ED7, ED14, and ED21 (*p* < 0.05). Compared to those in the CONT group, piglets in the IUGR group had much lower serum HOMA-IR on ED7, ED14, and ED21 (*p* < 0.05). Compared to those in the IUGR group, piglets in the I-LEU group had higher HOMA-IR on ED7, ED14 and ED21 (*p* < 0.05).

The serum leucine concentrations on ED21 are also shown in Table 4. Serum leucine concentrations on ED21 were significantly affected by birth weight (*p* < 0.05). The interaction between birth weight and leucine supplementation was significant for serum leucine concentrations on ED21 (*p* < 0.05). Compared to those in the CONT group, piglets in the IUGR group had lower serum leucine concentrations on ED21 (*p* < 0.05). Compared to those in the IUGR group, piglets in the I-LEU group had higher serum leucine concentrations (*p* < 0.05).

### 3.3. Effects of Leucine on Live Glycogen Concentration in IUGR Piglets

**Figure 1:**
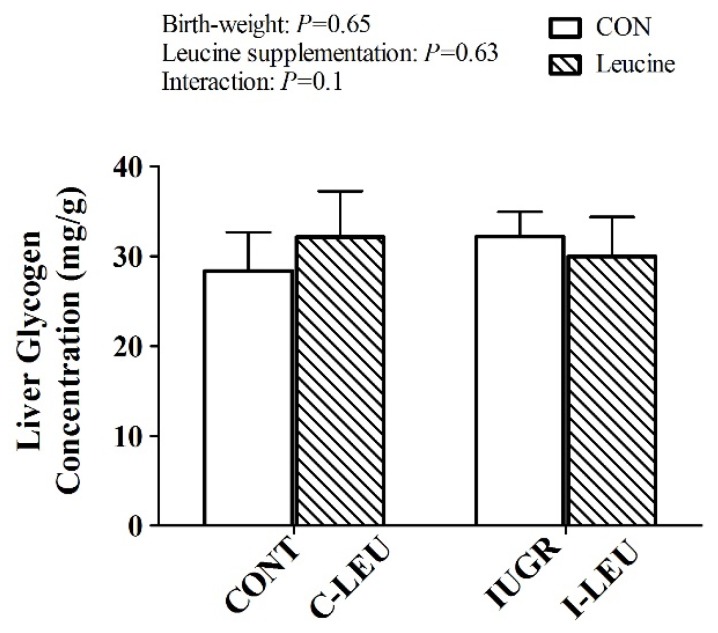
Effect of dietary leucine levels on liver glycogen in IUGR piglets. CONT, normal birth weight piglets fed a basal diet; C-LEU, normal birth weight piglets fed a basal diet with the addition of 0.35% l-leucine; IUGR, low birth weight piglets fed a basal diet; I-LEU, low birth weight piglets fed a basal diet with the addition of 0.35% l-leucine. Values are expressed as the mean ± SEM, n = 8 piglets per treatment. Within the same row, means with different superscripts are different at *p* < 0.05.

The liver glycogen concentration is shown in Figure 1. There is no significant effect of birth weight, leucine supplementation or their interaction on the liver glycogen concentration of piglets (*p* > 0.05).

### 3.4. Effects of Leucine on Serum Total Cholesterol, Triglyceride, and Creatinine Concentrations in IUGR Piglets

The serum total cholesterol, triglyceride, and creatinine concentrations are shown in Table 5. The interaction between birth weight and leucine supplementation was significant for serum triglyceride concentrations on ED14 (*p* < 0.05). There was no significant difference on serum triglyceride concentrations between the piglets in the IUGR and I-LEU group (*p* > 0.05). Serum creatinine concentrations on ED21 were significantly affected by birth weight (*p* < 0.05). The interaction between birth weight and leucine supplementation was significant for serum creatinine concentrations on ED21 (*p* < 0.05). Compared to those in the CONT group, piglets in the IUGR group had much lower serum creatinine concentrations (*p* < 0.05). Compared to those in the IUGR group, piglets in the I-LEU group had higher serum creatinine concentrations (*p* < 0.05).

### 3.5. Effects of Leucine on the Gene Expression of Insulin Signalling Pathway of Liver in IUGR Piglets

The gene expression of the insulin signaling pathway of liver in IUGR piglets is shown in Table 6. The gene expression of insulin receptor substrate 2 (*IRS-2*), phosphatidylinositol 3-kinase p110 gamma (*PIK3CG*), and protein kinase adenosine monophosphate-activated γ 3-subunit (*PRKAG3*) of liver were significantly affected by birth weight and leucine supplementation (*p* < 0.05). The interaction between birth weight and leucine supplementation was significant for the hepatic gene expression of *IRS-2*, *PIK3CG*, *PRKAG3*, and *IRS-1* (*p* < 0.05). Compared to those in the CONT group, piglets in the IUGR group had higher gene expression of *IRS-2* (*p* < 0.05). However, the gene expression of *IRS-2* in the piglets of the I-LEU group was not significantly different from that of those the IUGR group (*p* > 0.05). Compared to those in the IUGR group, piglets in the I-LEU group had higher gene expression of *PIK3CG* and *PRKAG3* (*p* < 0.05). The gene expression of *IRS-1* in the piglets of the IUGR group was higher than that of those in the CONT group (*p* < 0.05).

### 3.6. Effects of Leucine on the Gene Expression of Glucose Metabolism of Liver in IUGR Piglets

The gene expression of glucose metabolism of liver in IUGR piglets is shown in Table 7. The gene expression of glycogen synthase kinase-3 alpha (*GSK3A*) of liver was significantly affected by leucine supplementation (*p* < 0.05). The interaction between birth weight and leucine supplementation was significant for the hepatic gene expression of *GSK3A* (*p* < 0.05). Compared to those in the IUGR group, piglets in the I-LEU group had lower gene expression of *GSK3A* (*p* < 0.05). The gene expression of glucose transporter type 2 (*GLUT-2*) and *GLUT-4* of liver was significantly affected by birth weight and leucine supplementation (*p* < 0.05). The interaction between birth weight and leucine supplementation was significant for the hepatic gene expression of *GLUT-2* and *GLUT-4* (*p* < 0.05). Compared to those in the CONT group, piglets in the IUGR group had a lower gene expression of *GLUT-2* and *GLUT-4* (*p* < 0.05). Compared to those in the IUGR group, piglets in the I-LEU group had a higher gene expression of *GLUT-2* (*p* < 0.05).

## 4. Discussion

Early protein and energy intake of the newborns is associated with growth, development, and even resistance to various diseases. As for preterm or low birth weight infants, early postnatal nutrition is extremely vital. Data on neonatal piglets showed that shortage of protein and energy supply in the first week led to neurodevelopmental restriction in low birth weight infants [18]. Amino acid administration within 24 h after birth had a positive influence on 12-month developmental outcome and wellbeing of infants [28]. Valentine et al. found that early amino acid administration increased preterm infant weight [29]. The rate of weight gain is affected by energy intake, while changes in body size and head circumference were more attributed to protein intake during the early development of infants and newborn animals [30]. Thus, dietary nutrition interventions were suggested, in which sufficient protein intake and proper protein/energy ratio gained most of the attention. Our study showed that there was a positive relationship between the rate of weight gain and the intake of protein, fat, and energy. Dietary leucine supplementation decreased the weight loss of IUGR piglets after birth, suggesting its beneficial effect in growth promotion, as previous studies reported [25].

Our data showed that IUGR led to decreased glucose and insulin concentrations in serum. We also found IUGR significantly decreased insulin resistance through further calculating HOMA-IR. HOMA-IR has been widely utilized as an insulin resistance index in clinical studies [31]. Higher glucose and insulin levels, and HOMA-IR predict the increasing risk of insulin resistance [32]. However, the effects of IUGR on insulin resistance remain controversial. In suckling piglets [9] and adult rats [33], IUGR increased serum glucose and insulin concentrations and increased HOMA-IR, indicating the potential presence of insulin resistance. On the other hand, Ying et al. found that IUGR significantly decreased blood glucose and insulin level and HOMA-IR in piglets of 49 d [10]. Interestingly, Ying et al. also found that serum glucose and insulin concentrations tended to increase in piglets of 105 d, along with increased HOMA-IR [10]. Thus, we hypothesize that the effects of IUGR on insulin resistance are related to the age of piglets. Moreover, although increasing HOMA-IR reflects enhanced insulin resistance, we believe that HOMA-IR is indicative only within certain limits and HOMA-IR beyond the normal range could be explained as the abnormality of insulin metabolism. The extreme low values of HOMA-IR of IUGR piglets in the present study suggested that IUGR resulted in the disorder of systematic metabolism, especially insulin-related metabolism.

In the present study, leucine supplementation had no significant influences on serum glucose, insulin concentrations, and HOMA-IR in normal piglets, which was in accordance with previous findings [34]. Dietary leucine supplementation significantly increased serum glucose and insulin concentrations, and HOMA-IR in IUGR piglets. This was in agreement with the finding that leucine enhances the risk of developing insulin resistance [16,35]. Notably, serum glucose concentrations were increased by leucine supplementation in IUGR piglets and were recovered to the normal level compared to those in normal piglets. Serum insulin concentrations and HOMA-IR of piglets in the I-LEU group, while increased, were still significantly lower than those of the control group. Considering the abnormal response of IUGR, insulin resistance promoted by leucine actually alleviated the drastic changes of serum glucose and insulin concentrations induced by IUGR. The cause of IUGR and its effect on postnatal carbohydrate metabolism and organ function is complicated. It is unclear whether IUGR could influence serum insulin concentrations rather than interfering with the development and function of pancreas. In fact, leucine supplementation alleviated the abnormal changes of carbohydrate metabolism and insulin resistance induced by IUGR. Although leucine supplementation restored serum glucose concentrations and increased insulin concentrations in IUGR piglets, the insulin concentrations were still significantly lower than those in the control group. Increasing evidence has shown that elevated tissue and blood levels of leucine, both in obese humans and animals, prognosticate a future risk of developing insulin resistance and diabetes [35,36,37]. Therefore, we measured the serum leucine concentrations. Interestingly, leucine-induced elevation in the serum glucose and insulin concentrations was positively related to serum leucine concentrations, which was significantly increased in the I-LEU group.

Serum total cholesterol and triglyceride concentrations are a very important index in monitoring intracellular lipid metabolism. Ren et al. reported a marked decrease in serum total cholesterol and triglyceride concentrations with dietary leucine administration [38]. In our study, dietary leucine administration decreased serum total cholesterol and triglyceride concentrations in normal piglets. It might be related to either the depression of hepatic lipid synthesis or the enhanced elimination of triglyceride [39]. The increase of serum total cholesterol concentrations in IUGR piglets fed with leucine was possibly attributed to its effects on the ratio of phosphatidylcholine and phosphatidyl ethanolamine [40].

Liver plays an important role in the maintenance of glucose homeostasis. Infants mobilize adequate glucose in response to parturition stress and preweaning physiological metabolism [41]. The knockout or mutation of the above enzymes could result in the inhibition of glycogenesis and hypoglycemia [42]. Phosphoenolpyruvate carboxykinase (PEPCK) catalyzes the rate-limiting step in hepatic glucose synthesis, and its activity indicates glucose synthesis initiation in tissues [43]. Glycogen synthase 1 (GYS-1) catalyzes glycogen synthesis in skeletal muscle. G-6-P is highly expressed in liver and is a key enzyme in the regulation of blood glucose homoeostasis. G-6-P catalyzes the hydrolysis of glucose 6-phosphate derived from both gluconeogenesis and glycogenolysis and liberates the free glucose into circulation [44]. Lower serum glucose levels observed in preterm infants could be attributed to the inactivity of G-6-P [45]. Glycogen synthesis is limited to the depressed activity of the key enzyme, which is responsible for inefficient glucose production [46]. The present study showed that dietary leucine administration increased the gene expression of G-6-P in liver, suggesting leucine was likely to promote hepatic glycogen synthesis in IUGR piglets.

Animals showed a stable rate of glucose production around birth [43]. Weaning diet induces a major shift in physiological metabolism, including decreased hepatic gluconeogenesis, enhanced adipogenesis, and activated hepatic glucokinase (GCK) [47]. Hepatic GCK is an important key enzyme in glucose utilization. GCK facilitates phosphorylation of glucose to glucose-6-phosphate, which is the first step for both glycolysis and glycogen synthesis [48]. Jiang et al. found that the mRNA expression of GCK was positively correlated with hepatic glycogen concentrations [49]. These results are in accordance with our findings, stating that dietary leucine administration increased the mRNA expression level of *GCK* in IUGR piglets. However, leucine had no influence on hepatic glycogen concentration in the present study. We speculate there is a possible involvement of low insulin concentrations leading to reduced hepatic glycogen synthesis in IUGR piglets during the whole experimental period [50].

GLUT-2 is mainly expressed in the liver and mediates the bidirectional transport of glucose in cell plasma membrane. The upregulation of *GLUT-2* gene expression was involved in glucose output [51]. GLUT-4 is distributed intracellularly and facilitates glucose transport in muscle and fat cells. It was reported that the blood glucose levels of male rats were increased with the increasing gene and protein expression of GLUT-2 [52]. Our study showed that the gene expression of GLUT-2 was increased by the administration of leucine, along with the enhanced glucose uptake of liver in IUGR piglets. Based on the results of *G-6-P* and *GLUT-2* gene expression, we assumed that leucine facilitates glucose from liver transport in blood and peripheral tissue in IUGR piglets. The observed increase of blood glucose concentrations also confirmed our assumption in the I-LEU group.

The benefits of leucine in glycogen synthesis and glucose transport was likely associated with the insulin signaling pathway due to the presence of the increased *IRS-1* expression in the liver [53]. Previous studies reported that leucine stimulated the phosphorylation of IRS-1 through the mTOR pathway and led to the inhibition of the insulin signaling pathway [54]. Our results showed that dietary leucine supplementation decreased the gene expression of *IRS-1*. This reveals the improvement mediated by leucine in the insulin signaling pathway of IUGR piglets. Whether this paradox could be explained by the differences between normal and IUGR piglets or in vitro and in vivo needs to be further investigated.

Glucose transport is regulated by the initial and middle steps of the insulin signaling cascade, like the phosphorylation of the insulin receptor and IRS-1, and the PI3K/PKB/Akt signaling pathway [55]. Studies revealed an impaired insulin-stimulated phosphorylation of IRS-1 and a decrease of PI3K activity in the muscle from obese and type 2 diabetes [56,57]. IUGR newborns had low expression of the insulin signaling pathway in the muscle, including the phosphatidylinositol 3-kinase p110 gamma (PI3KCG), PRKAG, and protein kinase C epsilon zeta (PRKCZ) [58]. In IUGR piglets, there was a decreased phosphorylation of PRKCZ and Akt by insulin stimulation. Leucine administration decreased the expression of *PRKCZ*, suggesting the relationship of all these changes and a depressed glucose intake [59]. This demonstrated that leucine played a critical role in the regulation of the insulin signaling pathway in IUGR piglets.

## 5. Conclusions

In our study, IUGR adversely affected lipid metabolism and glucose metabolism in the liver. These adverse effects of IUGR were alleviated by leucine supplementation in piglets. Dietary leucine supplementation restored serum glucose, increased insulin and creatinine concentrations on ED21, and enhanced PRKAG3 and GLU-2 expression. These findings suggest that leucine might play a positive role in hepatic lipid metabolism and glucose metabolism in IUGR. Further studies are needed to investigate the potential effects of leucine on the complex network of genes involved in the insulin signaling pathway and glucose transporters due to the complexity of IUGR models.

## Figures and Tables

**Table 1:** The composition and nutrient content of the basal diets.

Items	Treatments			
	CONT	IUGR	C-LEU	I-LEU
Ingredients (%)				
Corn	55.00		55.00	
Soybean meal, fermented	15.00		15.00	
Soybean meal, de-hulled	6.00		6.00	
Whey powder	7.00		7.00	
Fish meal	4.00		4.00	
Glucose	7.50		7.50	
Soybean oil	1.50		1.50	
Leucine	0.00		0.35	
Limestone	1.20		1.20	
CaHPO_4_	0.80		0.80	
Premix ^1^	2.00		2.00	
Calculated analysis				
Digestible energy, (Mcal/kg)	3.40		3.40	
CP, %	20.20		20.20	
Lys, %	1.45		1.45	
Met+Cys, %	0.79		0.79	
Thr, %	0.81		0.81	
Leu, %	1.45		1.80	
Ca, %	0.85		0.85	
TP, %	0.70		0.70	

^1^ Provided per kg of diet: Vitamin A 15,000 IU; Vitamin B_1_ 3 mg; Vitamin B_2_ 6 mg; Vitamin B_6_ 5 mg; Vitamin B_12_ 0.03 mg; Vitamin C 250 mg; Vitamin D_3_ 3,000 IU; Vitamin E 150 mg; Vitamin K_3_ 3 mg; Niacin 45 mg; Calcium pantothenate 9 mg; Folic acid 1 mg; Biotin 0.3 mg; Choline chloride 500 mg; Fe 170 mg; Cu 150 mg; I 0.90 mg; Se 0.2 mg; Zn 150 mg; Mg 68 mg; Mn 80 mg; Co 0.30 mg.

**Table 2:** Primer sequences used in real-time quantitative PCR analysis.

Gene ^1^	Primer Sequence (5′–3′)	Product Length (bp)	GeneBank Accession No.
*AKT-2*	F:GGCAGCGACACGAGACTG	87	XM_013988563.1
	R:GACACGCTGTCACCTAGCTT		
*IRS-1*	F:GCCACGGGAGAATGGGTTTA	139	NM_001244489
	R:GTCGCACACAGTTTCAGCAG		
*IRS-2*	F:TTCCTCCTTCCGTGGTGAAC	130	GU722144.1
	R:TGCACTGTTGTGCTGTGTGA		
*PIK3CG*	F:CCTGAAGGATCCCAAAGCGT	120	NM_213939
	R:AGAAGTTGCAGTCCAGGAGC		
*PRKCZ*	F:TGCCCCGGAGAAGACAAATC	71	NM_001204374
	R:TATGCCTCTTGCAGGTCAGC		
*PRKAG3*	F:CAGATCTCAGACTGGGGCAC	96	NM_214077
	R:AAGGCACGTCACTGATCTGG		
*GCK*	F:GAGATGAGCAGGTACGGAGC	186	NM_001143708.1
	R:GCAGTGCAGACATGCAACAA		
*GYS-1*	F:AAACATGCAGCATCGGGGTA	121	NM_001195508.1
	R:TCCACAGCACCAGCACATAG		
*GSK3A*	F:AGCTGATCTTTGGAGCCACC	133	HM214804.1
	R:TCGAATCTGTTCCCGGGTTG		
*G-6-P*	F:CAGATCTACCGCATCGACCA	113	XM_003360515.4
	R:CAGGCGATGTTGTCTCGGTT		
*GLUT-2*	F:GACACGTTTTGGGTGTTCCG	105	NM_001097417
	R:GAGGCTAGCAGATGCCGTAG		
*GLUT-4*	F:CCAGCCTATGCTACCTAAGACA	147	EU590115.1
	R:AGAGAGCCCAGAGGGTAGTG		
*GAPDH*	F:ACTGAGGACCAGGTTGTGTC	71	NM_001206359
	R:CCAGCCCCAGCATCAAAAGT		

^1^*AKT-2*: protein kinase B 2; *IRS-1*: insulin receptor substrate 1; *IRS-2*, insulin receptor substrate 2; *PIK3CG*, phosphatidylinositol 3-kinase p110 gamma; *PRKCZ*, protein kinase C epsilon zeta; *PRKAG3*, protein kinase adenosine monophosphate-activated γ 3-subunit; *GCK*, glucokinase; *GYS-1*, glycogen synthase 1; *GSK3A*, glycogen synthase kinase-3 alpha; *G-6-P*, glucose-6-phosphatase; *GLUT-2*, glucose transporter type 2; *GLUT-4*, glucose transporter type 4; *GAPDH*, glyceraldehyde-3-phosphate dehydrogenase.

**Table 3:** Effect of leucine on growth performance in intrauterine growth restriction/retardation (IUGR) piglets.

Items	Treatments				SEM	*p*-Value		
	CONT ^1^	C-LEU	IUGR	I-LEU		Birth Weight	Leucine Supplementation	Interaction
0–21 d								
FBW ^2^, kg	9.79 ^a^	9.41 ^a^	6.18 ^c^	7.02 ^b^	0.29	0.002	0.244	0.003
BWG, kg	4.33 ^a^	3.98 ^ab^	2.74 ^c^	3.45 ^b^	0.78	0.008	0.250	0.003
Protein intake, g/d	68.23 ^a^	62.04 ^b^	48.92 ^c^	57.75 ^b^	8.25	0.006	0.400	0.002
Fat intake, g/d	15.20 ^a^	13.82 ^b^	10.90 ^c^	12.87 ^b^	1.84	0.005	0.400	0.002
Energy intake, kcal/d	1148.4 ^a^	1044.2 ^b^	823.38 ^c^	972.06 ^b^	138.9	0.006	0.400	0.003
BWG of each phase, kg								
0–7d	1.09 ^a^	0.83 ^b^	0.74 ^b^	0.84 ^b^	0.22	0.015	0.240	0.008
7–14d	1.80 ^a^	1.63 ^a^	1.05 ^b^	1.36 ^b^	0.33	0.003	0.730	0.017
14–21d	1.44	1.52	0.95	1.25	0.35	0.008	0.001	0.421

^1^ CONT, normal birth weight piglets fed a basal diet; C-LEU, normal birth weight piglets fed a basal diet with the addition of 0.35% L-leucine; IUGR, low birth weight piglets fed a basal diet; I-LEU, low birth weight piglets fed a basal diet with the addition of 0.35% L-leucine. ^2^ FBW, final body weight; BWG, body weight gain. ^a, b^ Values are expressed as the mean ± SEM, n = 8 piglets per treatment. Within the same row, means with different superscripts are different at *p* < 0.05.

**Table 4:** Effect of leucine on serum glucose, insulin, and leucine concentration in IUGR piglets.

Items	Treatments				SEM	*p*-Value		
	CONT ^1^	C-LEU	IUGR	I-LEU		Birth Weight	Leucine Supplementation	Interaction
Glucose, mmol/L								
ED0	5.59	5.65	4.30	4.47	0.70	0.002	0.390	0.670
ED7	5.53	6.07	4.72	5.42	0.54	0.003	0.003	0.490
ED14	5.33 ^a^	5.45 ^a^	4.30 ^b^	5.30 ^a^	0.58	0.002	0.002	0.008
ED21	6.27 ^b^	7.35 ^a^	4.59 ^c^	6.41 ^b^	1.06	0.002	0.005	0.004
Insulin, pmol/L								
ED0	66.96	67.71	48.2	47.91	10.18	0.002	0.850	0.660
ED7	86.08 ^a^	76.97 ^b^	49.64 ^c^	73.79 ^b^	13.64	0.009	0.003	0.001
ED14	96.29 ^a^	88.54 ^b^	57.5 ^d^	80.05 ^c^	14.99	0.003	0.007	0.004
ED21	99.33 ^a^	86.74 ^b^	58.9 ^c^	83.55 ^b^	15.14	0.007	0.037	0.005
HOMA-IR ^2^								
ED0	2.124	2.226	1.196	1.234	0.12	0.000	0.332	0.654
ED7	2.78 ^a^	2.686 ^a^	1.375 ^c^	2.324 ^b^	0.14	<0.001	0.001	<0.001
ED14	2.958 ^a^	2.772 ^a^	1.475 ^c^	2.444 ^b^	0.14	<0.001	0.001	<0.001
ED21	3.5667 ^a^	3.758 ^a^	1.59 ^c^	3.105 ^b^	0.18	<0.001	<0.001	<0.001
Leucine, μmol/L								
ED21	151.29 ^b^	161.00 ^b^	112.30 ^c^	205.50 ^a^	9.44	0.005	0.787	0.001

^1^ CONT, normal birth weight piglets fed a basal diet; C-LEU, normal birth weight piglets fed a basal diet with the addition of 0.35% L-leucine; IUGR, low birth weight piglets fed a basal diet; I-LEU, low birth weight piglets fed a basal diet with the addition of 0.35% L-leucine. ^a–c^ Values are expressed as the mean ± SEM, n = 8 piglets per treatment. Within the same row, means with different superscripts are different at *p* < 0.05. ^2^ Homeostasis models of assessment for the insulin resistance index (HOMA-IR) were calculated as follows: HOMA-IR = (fasting glucose (mmol/L) × fasting insulin (μU/mL))/22.5.

**Table 5:** Effect of leucine on serum total cholesterol, triglyceride, and creatinine concentrations in IUGR piglets.

Items	Treatments				SEM	*p*-Value		
	CONT ^1^	C-LEU	IUGR	I-LEU		Birth Weight	Leucine Supplementation	Interaction
Cholesterol, mmol/L								
ED0	3.60	3.83	3.33	3.31	0.12	0.120	0.670	0.620
ED7	2.02	2.10	1.81	1.92	0.06	0.020	0.240	0.840
ED14	1.62	1.72	1.54	2.03	0.08	0.450	0.060	0.200
ED21	1.85 ^a^	1.53 ^b^	1.59 ^b^	1.67 ^ab^	0.04	0.470	0.150	0.030
Triglyceride, mmol/L								
ED0	0.52	0.67	0.38	0.61	0.03	0.080	0.001	0.450
ED7	0.26	0.28	0.33	0.35	0.02	0.030	0.630	0.890
ED14	0.30 ^b^	0.46 ^a^	0.41 ^ab^	0.31 ^b^	0.02	0.690	0.450	0.003
ED21	0.65	0.51	0.61	0.64	0.03	0.400	0.320	0.130
Creatinine, μmol/L								
ED0	115.69	110.63	107.09	106.53	1.86	0.100	0.450	0.540
ED7	117.58	113.89	109.27	114.38	1.57	0.220	0.820	0.170
ED14	111.07	115.66	117.50	116.30	2.16	0.440	0.710	0.530
ED21	121.86 ^a^	113.02 ^a^	95.11 ^b^	111.70 ^a^	2.70	0.006	0.380	0.007

^1^ CONT, normal birth weight piglets fed a basal diet; C-LEU, normal birth weight piglets fed a basal diet with the addition of 0.35% L-leucine; IUGR, low birth weight piglets fed a basal diet; I-LEU, low birth weight piglets fed a basal diet with the addition of 0.35% L-leucine. ^a, b^ Values are expressed as the mean ± SEM, n = 8 piglets per treatment. Within the same row, means with different superscripts are different at *p* < 0.05.

**Table 6:** Effect of leucine on the gene expression of the insulin signaling pathway of liver in IUGR piglets.

Items	Treatments				SEM	*p*-Value		
	CONT ^1^	C-LEU	IUGR	I-LEU		Birth Weight	Leucine Supplementation	Interaction
*AKT-2* ^2^	1.00	0.82	0.89	1.11	0.04	0.380	0.840	0.050
*IRS-1*	1.00 ^b^	1.67 ^a^	1.73 ^a^	1.16 ^ab^	0.11	0.570	0.780	0.045
*IRS-2*	1.00 ^b^	3.76 ^a^	4.29 ^a^	4.18 ^a^	0.07	0.001	0.003	0.007
*PIK3CG*	1.00 ^c^	1.39 ^b^	1.00 ^c^	2.27 ^a^	0.12	0.003	0.006	0.004
*PRKCZ*	1.00	1.01	0.80	0.51	0.06	0.008	0.160	0.120
*PRKAG3*	1.00 ^b^	0.93 ^b^	1.25 ^b^	4.94 ^a^	0.40	0.001	0.007	0.003

^1^ CONT, normal birth weight piglets fed a basal diet; C-LEU, normal birth weight piglets fed a basal diet with the addition of 0.35% L-leucine; IUGR, low birth weight piglets fed a basal diet; I-LEU, low birth weight piglets fed a basal diet with the addition of 0.35% L-leucine. ^2^ *AKT-2*, Protein kinase B 2; *IRS-1*, Insulin receptor substrate 1; *IRS-2*, Insulin receptor substrate 2; *PIK3CG*, phosphatidylinositol 3-kinase p110 gamma; *PRKCZ*, protein kinase C epsilon zeta; *PRKAG3*, protein kinase adenosine monophosphate-activated γ 3-subunit. ^a, b^ Values are expressed as the mean ± SEM, n = 8 piglets per treatment. Within the same row, means with different superscripts are different at *p* < 0.05.

**Table 7:** Effect of leucine on the gene expression of glucose metabolism of liver in IUGR piglets.

Items	Treatments				SEM	*p*-Value		
	CONT ^1^	C-LEU	IUGR	I-LEU		Birth Weight	Leucine Supplementation	Interaction
*GCK* ^2^	1.00	2.49	1.31	2.73	0.19	0.260	0.009	0.910
*GYS-1*	1.00	1.52	0.86	1.23	0.07	0.030	0.006	0.440
*GSK3A*	1.00 ^b^	1.11 ^b^	1.12 ^b^	0.83 ^a^	0.03	0.110	0.060	0.006
*G-6-P*	1.00	1.38	0.43	1.59	0.08	0.450	0.008	0.120
*GLUT-2*	1.00 ^bc^	3.40 ^a^	0.30 ^c^	1.77 ^b^	0.33	0.001	0.007	0.009
*GLUT-4*	1.00 ^b^	1.91 ^a^	0.63 ^b^	0.55 ^b^	0.12	0.003	0.002	0.004

^1^ CONT, normal birth weight piglets fed a basal diet; C-LEU, normal birth weight piglets fed a basal diet with the addition of 0.35% L-leucine; IUGR, low birth weight piglets fed a basal diet; I-LEU, low birth weight piglets fed a basal diet with the addition of 0.35% L-leucine. ^2^ *GCK*, glucokinase; *GYS-1*, glycogen synthase 1; *GSK3A*, glycogen synthase kinase-3 alpha; *G-6-P*, glucose-6-phosphatase; *GLUT-2*, Glucose transporter type 2; *GLUT-4*, Glucose transporter type 4. ^a, b^ Values are expressed as the mean ± SEM, n = 8 piglets per treatment. Within the same row, means with different superscripts are different at *p* < 0.05.
